# Human Stefin B Role in Cell's Response to Misfolded Proteins and Autophagy

**DOI:** 10.1371/journal.pone.0102500

**Published:** 2014-07-21

**Authors:** Mira Polajnar, Tina Zavašnik-Bergant, Katja Škerget, Matej Vizovišek, Robert Vidmar, Marko Fonović, Nataša Kopitar-Jerala, Uroš Petrovič, Susanna Navarro, Salvador Ventura, Eva Žerovnik

**Affiliations:** 1 Department of Biochemistry and Molecular and Structural Biology, Jožef Stefan Institute, Ljubljana, Slovenia; 2 Jožef Stefan International Postgraduate School, Ljubljana, Slovenia; 3 Department of Molecular and Biomedical Science, Jožef Stefan Institute, Ljubljana, Slovenia; 4 Institute of Biotechnology and Biomedicine, Autonomous University of Barcelona, Bellaterra, Spain; 5 CipKeBip - Center of Excellence for integrated approaches in chemistry and biology of proteins, Ljubljana, Slovenia; University of Arkansas for Medical Sciences, United States of America

## Abstract

Alternative functions, apart from cathepsins inhibition, are being discovered for stefin B. Here, we investigate its role in vesicular trafficking and autophagy. Astrocytes isolated from stefin B knock-out (KO) mice exhibited an increased level of protein aggregates scattered throughout the cytoplasm. Addition of stefin B monomers or small oligomers to the cell medium reverted this phenotype, as imaged by confocal microscopy. To monitor the identity of proteins embedded within aggregates in wild type (wt) and KO cells, the insoluble cell lysate fractions were isolated and analyzed by mass spectrometry. Chaperones, tubulins, dyneins, and proteosomal components were detected in the insoluble fraction of wt cells but not in KO aggregates. In contrast, the insoluble fraction of KO cells exhibited increased levels of apolipoprotein E, fibronectin, clusterin, major prion protein, and serpins H1 and I2 and some proteins of lysosomal origin, such as cathepsin D and CD63, relative to wt astrocytes. Analysis of autophagy activity demonstrated that this pathway was less functional in KO astrocytes. In addition, synthetic dosage lethality (SDL) gene interactions analysis in *Saccharomyces cerevisiae* expressing human stefin B suggests a role in transport of vesicles and vacuoles These activities would contribute, directly or indirectly to completion of autophagy in wt astrocytes and would account for the accumulation of protein aggregates in KO cells, since autophagy is a key pathway for the clearance of intracellular protein aggregates.

## Introduction

Stefin B belongs to a superfamily of cystatins, cysteine protease inhibitors. It is localized both in the nucleus and in the cytoplasm where it inhibits activity of cathepsins B, H, K, L, and S [1]. Mutations in the stefin B (cystatin B) gene are the underlying cause of the progressive myoclonus epilepsy of type 1 (EPM1) [2], [3], with features of neurodegeneration [4], [5]. EPM1 belongs to larger group of progressive myclonus epilepsies (PMEs), a group of genetic generalized epilepsies with different etiologies. The most common mutation is a dodecamer repeat expansion in the promoter region of the gene found in 90% of patients that causes decreased mRNA and protein levels of stefin B [6]. Other EPM1 mutants translate to either truncated or missense proteins displaying different folding properties and different propensities to aggregate. We proposed in 2005 [7] that the prefibrillar oligomers of stefin B (and its aggregation-prone EPM1 mutants) might be toxic to cells, as it is the case for other amyloidogenic proteins. The cytotoxicity of the oligomers of stefin B and the aggregates of EPM1 mutants was later confirmed [8], [9]. *In vitro*, stefin B forms dimers, tetramers and higher-order oligomers, these latter assemblies being the cytotoxic species [10]. Domain-swapped dimers are believed to constitute the building blocks for fibril formation *in vitro* [11]. Accordingly, tetramers are formed by association of two domain-swapped dimers [12]. [13] [10], [12]Isolated higher-order oligomers bind to lipid monolayers effectively and are internalized through the plasma membrane via endocytosis, which results in decreased cell viability [8], [9], [10], [14]. It was shown that the wild-type (wt) stefin B forms oligomers, including trimers, also in cells [15]. Moreover, we have shown that wt stefin B and especially the missense and truncated EPM1 mutants form intracellular aggregates [16]. The endogenous protein formed small oligomers whereas wt stefin B and its EPM1 mutants all aggregated upon over expression. As expected, wt aggregated to a lower extent than disease-linked mutants.

The physiological role of human stefin B was until recently believed to be restricted to inhibition of the cysteine proteases (cathepsins), accidentally leaking from the lysosome. However, recent data show that stefin B also binds to histones and indirectly regulates the cell cycle through inhibition of cathepsin L in the nucleus [17]. Furthermore, additional and alternative functions in maintenance of cell homeostasis are being discovered including an amateur chaperone activity, binding to peptides such as Aβ [13], [18], SOD1 [19], reduction of oxidative stress [20], [21] and prevention of apoptosis [22], [23], [24]. In addition, due to its functional similarities with cystatin C [25], it has been proposed that stefin B might play a role in the induction of autophagy [26]. [25]

Autophagy has been described as non-selective degradative pathway induced by starvation, however, it plays additional roles than just nutrient management [27]. One of the main functions of autophagy is to enforce intracellular quality control by selective disposal of protein aggregates and damaged organelles (for review see [28]). Such an activity is best illustrated by the finding that neural-specific ablation of *atg5* and *atg7*, two essential genes for autophagy, leads to accumulation of ubiquitin-positive aggregates and progressive loss of neurons in mice [29], [30].

Autophagy starts with engulfing a portion of the cytoplasm surrounded by an isolation membrane into a cup-shaped phagophore, eventually forming a new vacuole known as an autophagosome [31] which next fuses with the lysosome. This step can be disrupted by dysfunction in the lysosomal pathway and/or by the susceptibility of the lysosome to oxidative stress generated by mitochondrial dysfunction or other cellular defects, including excessive protein aggregation. It is known that amyloid forming proteins in their soluble oligomeric forms can disrupt membrane integrity and even make pores, resembling bacterial pore-forming toxins (for review see [32], [33]).

Cystatin C induces autophagy via mTOR inhibition and thus acts as a pro-survival protein under stress conditions for the cell [25]. Cystatin C also has diverse functions, the best known is inhibition of cysteine proteases such as different cathepsins [34], [35] and mammalian legumain [36]. Cystatin C could exert a neuroprotective function by either preventing cell death, or promoting cell survival and neurogenesis. Indeed, cystatin C monomer was shown to interact with Aβ and inhibits *in vitro* Aβ fibril formation [37]. Of interest, stefin B interacted with Aβ in oligomer dependent way, namely, stefin B tetramers and a dimeric Y31 variant exhibited complete inhibition of amyloid formation by Aβ *in vitro* [13], [18]. Using electron-spray ionization mass spectrometry (ESI MS), it was shown that Aβ and stefin B Y31 variant dimer bind in a ratio of 1∶2, which means that one molecule of Aβ binds to a stefin B domain-swapped dimer and two molecules of Aβ to a stefin B tetramer [12] composed of two such dimers. Both cystatins might thus be neuroprotective by an amateur chaperone action.

Recently, neuronal cytoplasmic and intranuclear inclusions, containing the lysosomal protein cathepsin B and transmembrane protein CD68 (Cluster of Differentiation 68) or RNA-binding FUS (Fused in Sarcoma), respectively, were identified in one EPM1 patient [38] – as a consequence of stefin B deficiency. Therefore, similarly to cystatin C, stefin B could potentially induce autophagy and its absence would impair this process, leading to the accumulation of non processed protein aggregates. On the other hand, it was shown recently that absence of stefin B increases autophagy in a mouse model of Alzheimer's disease (AD) crossbred with stefin B KO mice [39]. These conflicting results demand additional studies [40].

In this study of wt and KO mice astrocytes, we aim to show how stefin B contributes to decrease protein aggregates and augments autophagy. A battery of methods was used: immunoblotting was used to trace autophagic markers, whereas protein aggregation was imaged by both confocal and electron microscopy. Mass spectroscopy was employed to identify the proteins embedded in the aggregates. We also analysed if this new function is dependent on stefin B oligomerization and/or cathepsins inhibition activity. In addition, we provide support to the data obtained on astrocytes by performing a synthetic dosage lethality (SDL) analysis in yeast cells expressing stefin B. Altogether, our results strongly suggest that stefin B could have a crucial role in chaperone and clearance pathways of misfolded proteins and their aggregated assemblies.

## Materials and Methods

### Ethics statement

Primary astrocytes were isolated from FVB wild-type and stefin B knock-out mice. No human or primates samples were used, neither experiments on humans performed. Permissions were obtained from the Veterinary Administration of the Republic of Slovenia (VURS) to work on animal (mice) tissue and mural cellular cultures. We sacrificed 30 FVB mice with knock-out stefin B gene (permission No 34401-11/2012/2 Date: 29.02.2012) and 30 wild type FVB mice (permission No 34401-9/2012/2, date 29.02.2012). All animals were sacrificed with cervical dislocation and bleeding out.

### Materials

Primers, *N*-α-benzoyl-DL-arginine β-naphthylamide substrate (BANA) (B4750), Fast Garnet GBS sulphate salt (F8761), paraformaldehyde (P6148), methyl cellulose (M7140), protease inhibitor cocktail (P8340), anti-rabbit IgG (A8275) and anti-mouse IgG (A9044) horseradish peroxidase-conjugated secondary antibodies were purchased from Sigma Aldrich (St. Louis, MO, USA). Amersham ECL Prime Western Blotting detection reagent was from GE Healthcare (RPN2232) (GE Healthcare Bio-Sciences AB, Uppsala, Sweden). E-64d was supplied by Bachem (N-1650) (Bubendorf, Switzerland). Lipofectamine 2000 (11668019), Prolong Antifade reagent with DAPI (P36931), and Bodipy 581/591 C_11_ (D3861) were from Invitrogen (Carlsbad, CA, USA). Prestained protein standard was used for sodium dodecyl sulphate polyacrylamide gel electrophoresis (SDS-PAGE) and Pfu DNA polymerase for the polymerase chain reaction (PCR) (#26619 and #EP0501, Fermentas, Vilnius, Lithuania). Gelatin (1040781000) and sucrose (573113) were from Merck (Whitehouse Station, NJ, USA). Uranyl acetate was purchased from SPI Supplies (West Chester, PA, USA). Sequencing grade modified trypsin (V5117) and 12% precast gels (59509) for LC-MS/MS analysis were purchased from Promega Corporation (Fitchburg, WI, USA) and Lonza (Basel, Switzerland), respectively.

For cell culture experiments, Dulbecco's modified Eagle's medium with high glucose and L-glutamine (E15-810), heat inactivated fetal bovine serum (FBS) (A15-104), penicillin/streptomycin (100×) (P11-010), Dulbecco's phosphate buffer saline (PBS) (H15-001), L-glutamine (M11-004), trypsin EDTA (L11-658, all PAA Laboratories GmbH, Pasching, Austria), and TrypLE Select (0040090DG, Gibco, Invitrogen, Carlsbad, CA, USA) were used. The following primary antibodies were used: anti-actin (A3852, Sigma Aldrich, St. Louis, MO, USA), anti-LC3 (ab78078 and ab58610, Abcam, Cambridge, UK) and anti-p62 (MABC32, Millipore, Millipore, Billerica, MA, USA). ProteoStat Protein aggregation assay kit was purchased from Enzo Life Sciences (ENZ-51023-KP002, Farmingdale, NY, USA).

### Cloning

For bimolecular fluorescence complementation (BiFC) stefin B gene (C3E31) (NM_000100.2) was inserted in a pcDNA4 vector (V1020-20, Invitrogen, Carlsbad, CA, USA) via Xho*I* and Xba*I* (R0146S and R0145S, New England Biolabs, Ipswich, MA, USA) restriction sites. Stefin B gene was additionally fused on its 3′ site with N-terminal or C-terminal halves of the yellow fluorescent protein (YFP). YFP halves were inserted via Xba*I* and Apa*I* (R0114S, New England Biolabs) restriction sites. For the design of the G4R mutant the following primers were used: 5′-CGAGGTCATGATGTGCCGGGCGCCCTCCGCCAC-3' and 5' GTGGCGGAGGGCGCCCGGCACATCATGACCTCG-3'.

DNA sequences were confirmed using Sanger sequencing (BigDye terminator kit) and resolved with Automatic Sequencer 3730XL (Applied Biosystems, Foster City, CA, USA) in Macrogen (Rockville, MD, USA).

### Protein expression in Escherichia coli (E. coli) and purification

Stefin B wt and G4R mutant were produced in *E. coli* and purified according to published procedures [41]. The proteins were additionally purified on the size exclusion chromatography using a Superdex 75 column in 0.01 M phosphate buffer, 0.12 M NaCl, pH 6.1. Stefin B was eluted as a set of well-defined oligomers, allowing isolation of monomers, dimers, tetramers, and higher oligomers. All of the recombinant proteins have Ser at position 3 instead of Cys to prevent covalent disulfide bond formation [13].

### BANA test

To evaluate the inhibitory activity of stefin, BANA test was performed. Stefin B monomers, dimers, tetramers and oligomers were diluted in BANA buffer (0.1 M phosphate buffer, 1.5 mM EDTA, pH 6.0) so that A_280_ was 0.5 (112 µM). Papain was diluted in the same buffer to 0.02 mg/ml (0.5 µM). Eight different molar ratios [E]∶[I] were prepared – 1∶22, 1∶11, 1∶4, 1∶2, 1∶0.2. Firstly, papain was activated with 5 mM cysteine for 5 minutes at 37°C. Next, BANA substrate was added to the reaction mixture in 2 mM final concentration and incubated for 10 minutes at 37°C. The reaction was stopped with a stop reagent (1 volume of reagent III∶1 volume of color reagent) and incubated at room temperature. Absorbance was measured at 520 nm on a Lambda 18 UV/VIS spectrometer (Perkin-Elmer, Waltham, MA, USA). Reagent III consisted of 10 mM p-chloromercurybenzoic acid and 50 mM EDTA at pH 6.0. Color reagent consisted of 3 mM Fast Garnet GBC salt in 4% Brij 35, pH 6.0.

### Cell culture and transient transfections

HEK293 cell line was grown in DMEM supplemented with high glucose and L-glutamine, 10% (v/v) FBS, 1% (v/v) penicillin/streptomycin at 37°C in 5% CO_2_. Cells were grown in 3 cm Petri dishes with a glass bottom (MatTek, Ashland, MA, USA) and transiently transfected using Lipofectamine 2000, according to the manufacturer's instructions.

### Bimolecular fluorescence (BiFC)

BiFC allows imaging of selective stable or transient protein interactions by reconstitution of, in our case, the split yellow fluorescent protein (YFP) [42]. Namely, upon interaction of the desired proteins, N-terminal (NYFP) and C-terminal (CYFP) halves bind together and produce a fluorescent protein. To discard any unspecific interactions, the one YFP half was fused with stefin B and the other with SUMO protein.

### Isolation of primary astrocytes

FVB wt and KO mice, between 50 and 70 weeks old, were selected for isolation of primary astrocytes from brain. KO mice were generated as described in [21]. The brain of the mice were taken out from the skull and cortex and hippocampus were cut out and transferred to a Petri dish with selective medium (L-15, 1% (v/v) GlutaMAX, 0.05% (v/v) penicillin/streptomycin, 0.1% (w/v) BSA). After centrifugation and changing the selective medium, samples were ground via suction through different size needles (0.8 mm, 0.6 mm and 0.45 mm) and finally filtered through 80 µm filters. Cells were centrifuged for 5 minutes at 1000×g and grown in DMEM supplemented with high glucose and L-glutamine, 20% (v/v) FBS, 1% (v/v) penicillin/streptomycin at 37°C in 5% CO_2_. The medium was changed every 3–4 days until the cells were at least 40% confluent. Then, cells were agitated twice over night and the medium was changed the next day.

### Cell lysates

For protein analysis, primary cells were lysed with radioimmunoprecipitation assay (RIPA) buffer (50 mM Tris–HCl, pH 7.4, 150 mM NaCl, 1% deoxycholate, 1% Triton X-100). Cells were scrapped and left on ice for 30 minutes. Cell lysates were clarified by centrifugation at 10000×g for 20 min at 4°C. For certain immunoblot analysis of cell lysates, cells were beforehand incubated with 80 nM bafilomycin for 30 minutes. Protein concentrations of the supernatants were determined by Bradford assay (500–0201, Bio-Rad Laboratories, Hercules, CA, USA)) and the absorbance was measured at 595 nm using an automatic plate reader.

For the induction of autophagy and inhibition of cathepsins, 20 nM rapamycin and 5 µM E-64d were added to the cells for 24 hours, respectively.

### Western blotting

Proteins were separated on 10% or 15% SDS–PAGE and transferred to nitrocellulose membranes in 192 mM glycine, 25 mM Tris, and 20% (v/v) methanol buffer. The membrane was blocked by incubating in Tris-buffered saline (TBS) buffer (pH 7.6) containing 5% (wt/vol) non-fat dry milk and sequentially incubated with primary antibodies. The membranes were incubated with horseradish-peroxidase conjugated secondary anti-mouse or anti-rabbit antibody (depending on the primary antibody). Proteins were visualized with ECL reagent according to the manufacturer's instructions. Luminescent signal of the bands on the membranes was captured using a Konica-Minolta SRX-101A dark box (Marunouchi, Chiyoda, Tokyo, Japan) and evaluated by ImageJ (National Institutes of Health NIH, Bethesda, Maryland, USA).

### Protein aggregates *ex vivo*

Presence of protein aggregates in primary astrocytes was observed under fluorescent confocal microscope and stained with Proteostat according to the manufacturer's instructions.

### Confocal fluorescence microscopy

Confocal images were taken at the Center of Excellence for Nanoscience and Nanotechnology (Department of Biochemistry, Molecular and Structural Biology, Jožef Stefan Institute, Ljubljana, Slovenia) or at the Microscopy Service of the Autonomous University of Barcelona (Edifici C Campus UAB, Cerdanyola del Vallés, Barcelona, Spain). Leica TCS SP5 X confocal microscope (Leica Microsystems GmbH, Wetzlar, Germany) was used for optical slicing (oil objective ×60, NA = 0.5). Proteostat was excited at 500 nm with white light laser (WLL) and DAPI was excited at 405 nm with an ultraviolet (UV) laser. For the BiFC experiments, YFP was excited with an Argon multilineal laser at 488 nm in live adherent cells. The emission signal was followed with a photomultiplier detector (PMT) or a hybrid detector (HyD). Leica LAS AF software (Leica Microsystems GmbH, Wetzlar, Germany) was used for image analysis.

### Transmission electron microscopy (TEM)

Cells were fixed with 4% paraformaldehyde at room temperature for 2 h. Mixture of growth medium and fixative was removed with a series of centrifugation steps and the pellet of cells embedded in 10% gelatin. Gelatin was solidified on ice and cells excised and cut into small blocks (1 mm^3^). Blocks of cells were then immersed in 2.6 M sucrose (cryoprotectant) in 0.1 M phosphate buffer and infiltrated at 4°C overnight. Finally, cryoprotected cells were mounted on aluminum specimen carriers and stored in liquid nitrogen. Trimming and sectioning of frozen blocks were performed on Leica EM FC6 ultramicrotome (Leica Microsystems GmbH, Wetzlar, Germany) for cryosectioning. Frozen specimen blocks were trimmed at −90°C and ultrathin cryosections (70 nm) were cut at −120°C using diamond 35° angle cryo-immuno knife (Diatome, Biel, Switzerland). Sections were collected and thawed on droplets of pick-up solution, i.e. 1∶1 mixture of 2.3 M sucrose and 2% methyl cellulose. Retrieved ultrathin sections of cells were put on carbon-coated Formvar film spread over copper grids (Agar Scientific, Elektron Technology UK Ltd., Essex, UK). Prior to uranyl acetate staining gelatin was removed from sections and grids rinsed on droplets of ultra-pure water. Sections were contrasted with 0.4% uranyl acetate in solution with 1.8% methyl cellulose (pH 4). Uranyl contrasted the cell proteins but not the lipids so the membranes appeared white on TEM micrographs. Contrasted cells were observed at 150 kV with JEM-2100 LaB6 transmission electron microscope (Jeol Ltd., Tokyo, Japan) at the Centre for Electron Microscopy, Jožef Stefan Institute, Ljubljana, Slovenia. Micrographs were taken using Gatan digital camera and DigitalMicrograph software (Gatan, Inc., Pleasanton, CA, USA).

### Oxidative stress

Oxidative stress in primary astrocytes was determined by Bodipy 2 µM 581/591 C_11_. Bodipy 581/591 C_11_ exhibits a shift in fluorescence emission peak from ∼590 nm to ∼510 nm after interaction with peroxyl radicals. This shift in fluorescence was detected by using flow cytometer. Primary astrocytes were washed with warm PBS, loaded with Bodipy 581/591 C_11_ in PBS for 30 minutes at 37°C. Cells were harvested, pelleted and resuspended in PBS and subjected to flow cytometry analysis. Dyes were excited using a 488 nm Ar laser and detected with the FL1 (515∼545 nm) detector on FACSCalibur (BD Biosciences, Franklin Lakes, NJ, USA) using CellQuest software (version 3.3). At least 7000 cells were acquired for each measurement with three or four parallels. For testing H_2_O_2_ sensibility, cells were incubated with 1 mM H_2_O_2_ for 30 minutes.

### Extraction of detergent-insoluble proteins

The insoluble fraction of cell lysates was isolated as described in [43].

### Sample preparation and analysis with mass spectrometer

Samples were separated on a 12.5% precast gel and stained with 0.1% Coomassie R 350. Protein lanes were cut into six slices and prepared for liquid chromatography-mass spectrometry (LC-MS/MS) analysis. The gel slices were destained, reduced by 10 mM DTT and alkylated by 55 mM iodoacetamide in 25 mM ammonium bicarbonate buffer pH 7.8. The proteins were digested with sequencing grade modified trypsin overnight at 37°C. The next day, extracted peptides were analyzed with Orbitrap LTQ Velos mass spectrometer (Thermo Scientific, Waltham, MA, USA), coupled to a nanoLC HPLC unit (Proxeon, Thermo Scientific, Waltham, MA, USA). Peptides were loaded on a C_18_ trapping column (Proxeon EASY-Column) and separated on a C18 PicoFrit AQUASIL analytical column (New Objective, Woburn, MA, USA). Mobile phase A (0.1% formic acid) was used for loading. Gradient 5–40% of mobile phase B (100% acetonitrile, 0.1% formic acid) was used for peptide separation. Overall flow rate was 300 nl/min. MS/MS spectra were obtained using collision-induced dissociation (CID) fragmentation. Database searches were performed in a mouse database (www.uniprot.org) using the MaxQuant software [44], [45]. Carbamidomethylation of cysteines (+57.02 Da) was set as fixed and methionine oxidation (+15.99 Da) as variable modification. Relative quantification was performed by spectral counting. Mass spectra were recorded twice for the wt and three times for the KO sample.

### Statistical analyses

For statistical analyses Office Analysis ToolPak was used employing Student's t-test: two samples assuming unequal variances. Graphs represent the mean and s.e.m. of at least three independent experiments unless otherwise stated.

### Synthetic dosage lethality screen (SDL)

SDL screening was performed as described in [46], using the custom-made robotic manipulator with 384 floating pin replicator.

## Results

### Increased amount of protein aggregates and oxidative stress in stefin B KO astrocytes

**Figure 1:**
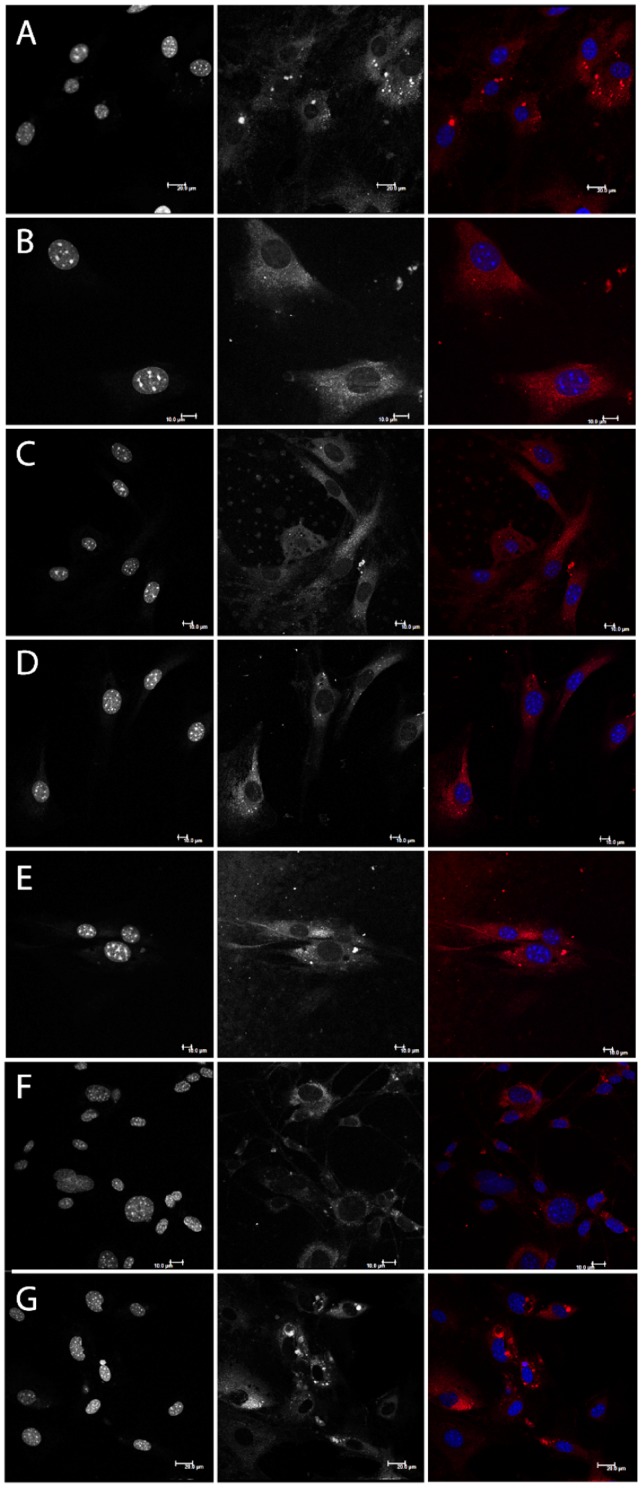
Fluorescence images of stefin B deficient (KO) and wild type (wt) astrocytes isolated from FVB mice stained with Proteostat and DAPI. KO cells were incubated with 30 µM stefin B proteins or 20 nM rapamycin for 24 hours. A: KO astrocytes control, B: KO astrocytes + monomers, C: KO astrocytes + dimers, D: KO astrocytes + tetramers, E: KO astrocytes + oligomers, F: wt astrocytes, G: KO astrocytes + rapamycin. Scale bar: 10 µm (except G: 20 µm).

**Figure 2:**
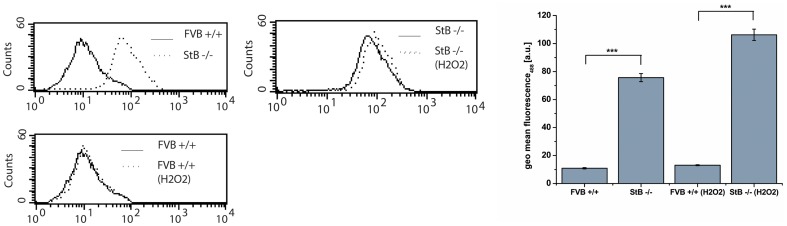
Shift in Bodipy 581/591 C_11_ fluorescence measured by flow cytometer. Bodipy 581/591 C_11_ was excited using a 488 nm Ar-laser and detected on a FL1 photodetector (515∼545 nm) on flow cytometer. Untreated FVB +/+ wild type (wt) astrocytes had lower lipid peroxidation signal compared to StB −/− knock out (KO) cells. KO cells were more sensitive to 1 mM H_2_O_2_ (30′) treatment. The representative results of one experiment (out of three) are shown.

Increased protein aggregation levels in stefin B KO astrocytes, relative to wt cells, could be clearly visualized upon staining with Proteostat dye (Figure 1). [16]We examined the impact of aggregation in oxidative stress by measuring lipid peroxidation in KO and wt astrocytes. KO cells showed increased oxidative stress levels and were also more sensitive to treatment with H_2_O_2_ (Figure 2).

As aggregates are considered to be mostly cleared via autophagy [16], we presumed that their levels will be reduced after treating KO astrocytes with an autophagy inducer. We used rapamycin, an inhibitor of the mTOR kinase in the mTOR complex through which autophagy is induced. However, rapamycin did not reduce the presence of aggregates (Figure 1G). In agreement, in an independent study on HEK293T cells overexpressing EPM1 mutants, rapamycin promoted aggregation (our unpublished data). To assess whether wt stefin B monomers, dimers and tetramers could have a function in aggregate clearance, the KO astrocytes were incubated with isolated stefin B monomers, dimers, tetramers and higher oligomers [10] for 24 hours. Intracellular protein aggregates got reduced upon addition of stefin B monomers, dimers and tetramers (Figure 1B-E). The effect was less obvious for higher-order oligomers, thus suggesting that this novel function might depend on the protein's inhibitory activity.

The inhibitory activity of monomers and isolated oligomers, i.e. dimers, tetramers and higher oligomers was tested using the BANA test (Figure S1). All oligomeric forms kept their inhibitory activity but exhibited an approximately 2-fold lower inhibition of papain in comparison to monomers. Dimers, and tetramers were at least 95% pure, therefore, we could exclude their activity being caused by a major monomer contamination. The mixture of the higher-order oligomers was less pure and its inhibitory activity could possibly be ascribed to contamination with an amount of monomers and lower-order oligomers and may vary depending on the composition of the mixture.

### Characterization of protein aggregates in stefin B astrocytes by TEM

**Figure 3:**
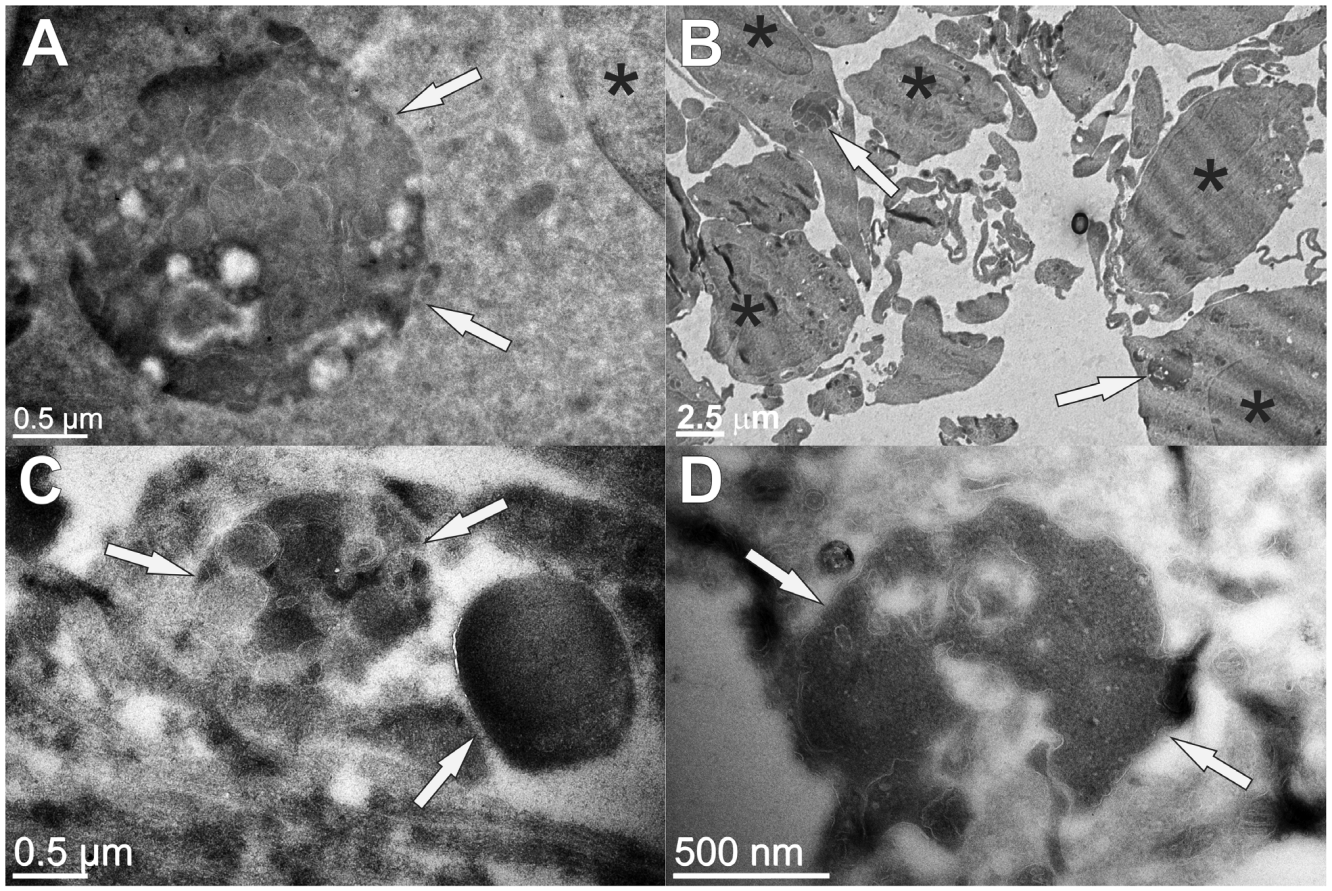
Transmission electron microscopy (TEM) of wild type (A, B) and KO (C, D) astrocytes from FVB mice. On ultrathin sections (70 nm) cell proteins were contrasted with uranyl acetate, whereas membranes remained white (not contrasted). The positions of large complex aggresome-like structures with high protein content and various membrane inclusions (*arrows*) and the position of nuclei (*) are denoted. The measured diameter of marked electron-dense structures (*arrows*) is 2.4 µm (A), 1.5 µm (C) and 1.9 µm (D), respectively, and their position in cells with regard to the cell nuclei is displayed at lower magnification (B).

The protein inclusions were visualized by transmission electron microscopy (TEM). In Figure 3, comparison between the wt and KO astrocytes is shown. Large complex structures strongly contrasted with uranyl acetate could be observed, indicating their elevated protein content. Various membrane inclusions were also observed and these big structures were more abundant and show more complex composition in the wt astrocytes (Figure 3A, B, Figure S2A) than in KO cells (Figure 3C, D). Their average size in the wt astrocytes was larger with diameters between 2 µm and 3 µm (Figure 3A), compared to their KO counterparts with diameters lower than 2 µm (Figure 3C, D).

### Characterization of protein aggregates in stefin B astrocytes by mass spectrometry

In order to identify the content of the protein inclusions, LC-MS/MS analysis of the insoluble fraction of wt and KO astrocytes lysates was performed (Table 1 – extracted from Table S1). Some constituents of the insoluble structures in KO cells were of lysosomal origin (cathepsin D and CD63). Apolipoprotein E and major prion protein, typically associated with Alzheimer's and prion's disease respectively, were detected only in the aggregates of KO cells. The KO astrocytes also exhibited an increased level of serpins H1 and I2, serine protease inhibitors and of fibronectin. On the other hand, chaperones and proteins of the ubiquitin proteasome system were decreased or not detected at all in the insoluble fraction of KO cells. The only chaperone found in KO cells was clusterin, a protein involved in prevention of stress-induced aggregation (Table 1).

**Table 1:** Selected proteins identified by LC-MS/MS analysis from the insoluble fraction of wild type (WT) or stefin B knock out (KO) astrocytes.

Protein IDs	Protein names	Gene names	PEP	MS/MS Count KO	MS/MS Count WT
**P08226**	**Apolipoprotein E**	**Apoe**	**1,56E-28**	**8**	**0**
**P18242**	**Cathepsin D**	**Ctsd**	**4,07E-15**	**9**	**1**
**P41731**	**CD63 antigen**	**Cd63**	**2,87E-16**	**8**	**4**
**Q06890**	**Clusterin**	**Clu**	**8,48E-38**	**6**	**0**
O08788	Dynactin subunit 1	Dctn1	1,31E-26	1	9
Q99KJ8	Dynactin subunit 2	Dctn2	2,22E-23	2	8
Q9Z0Y1	Dynactin subunit 3	Dctn3	1,09E-16	0	2
Q8K1M6	Dynamin-1-like protein	Dnm1l	7,45E-06	0	2
P39054	Dynamin-2	Dnm2	5,10E-21	0	8
P63168	Dynein light chain 1, cytoplasmic	Dynll1	3,23E-67	3	11
P51807	Dynein light chain Tctex-type 1	Dynlt1	1,89E-166	0	4
**P11276**	**Fibronectin**	**Fn1**	**0**	**1119**	**10**
P17879	Heat shock 70 kDa protein 1B	Hspa1b	1,38E-65	0	10
Q61316	Heat shock 70 kDa protein 4	Hspa4	2,61E-107	0	11
P07901	Heat shock protein HSP 90-alpha	Hsp90aa1	4,37E-277	10	75
P04925	Major prion protein	Prnp	1,33E-06	3	0
P62334	26S protease regulatory subunit 10B	Psmc6	5,73E-45	2	24
P62192	26S protease regulatory subunit 4	Psmc1	7,84E-73	0	13
O88685	26S protease regulatory subunit 6A	Psmc3	3,55E-93	0	16
P54775	26S protease regulatory subunit 6B	Psmc4	4,87E-44	0	12
P46471	26S protease regulatory subunit 7	Psmc2	3,05E-24	1	12
P62196	26S protease regulatory subunit 8	Psmc5	3,74E-14	1	12
Q3TXS7	26S proteasome non-ATPase regulatory subunit 1	Psmd1	2,04E-37	0	11
Q8BG32	26S proteasome non-ATPase regulatory subunit 11	Psmd11	3,03E-28	0	15
Q9D8W5	26S proteasome non-ATPase regulatory subunit 12	Psmd12	1,12E-81	2	14
Q9WVJ2	26S proteasome non-ATPase regulatory subunit 13	Psmd13	2,96E-15	0	7
O35593	26S proteasome non-ATPase regulatory subunit 14	Psmd14	2,77E-43	0	8
Q8VDM4	26S proteasome non-ATPase regulatory subunit 2	Psmd2	1,13E-128	0	27
P26516	26S proteasome non-ATPase regulatory subunit 7	Psmd7	3,05E-64	2	11
Q9Z2U1	Proteasome subunit alpha type-5	Psma5	4,94E-64	0	8
Q9CWH6	Proteasome subunit alpha type-7-like	Psma8	1,76E-16	0	6
O09061	Proteasome subunit beta type-1	Psmb1	2,28E-08	0	4
P99026	Proteasome subunit beta type-4	Psmb4	3,54E-33	0	5
O55234	Proteasome subunit beta type-5	Psmb5	6,50E-16	0	6
Q60692	Proteasome subunit beta type-6	Psmb6	4,96E-16	0	4
Q6PDI5	Proteasome-associated protein ECM29 homolog	Ecm29	3,09E-58	0	10
**P19324**	**Serpin H1**	**Serpinh1**	**2,78E-58**	**14**	**0**
**Q9JK88**	**Serpin I2**	**Serpini2**	**2,31E-05**	**6**	**0**
Q9JMA1	Ubiquitin carboxyl-terminal hydrolase 14	Usp14	2,27E-08	0	5
P56399	Ubiquitin carboxyl-terminal hydrolase 5	Usp5	1,71E-117	1	19
Q9JKB1	Ubiquitin carboxyl-terminal hydrolase isozyme L3	Uchl3	1,48E-89	1	8
Q9WUP7	Ubiquitin carboxyl-terminal hydrolase isozyme L5	Uchl5	3,63E-13	0	5
Q80×50-5	Ubiquitin-associated protein 2-like	Ubap2l	1,68E-20	0	8
Q922F4	Tubulin beta-6 chain	Tubb6	1,25E-289	1	26
P83887	Tubulin gamma-1 chain	Tubg1	2,02E-18	2	8

Proteins stressed **in bold** were increased in stefin B KO cells. Database searches were performed on a mouse database (www.uniprot.org) using the MaxQuant software [44], [45]. Relative label-free quantification (LTQ) was performed by spectral counting. Mass spectra were recorded twice for the wild type and three times for the knock out sample. Results of one experiment are shown. Posterior error probability (PEP) is the probability of a false hit given the peptide identification and length of peptides. MS/MS count column represents the number of recorded spectra.

### Evaluating autophagic flux in stefin B KO cells

LC3 and p62/SQSTM are commonly used marker proteins to evaluate autophagy in cells [47], [48]. p62 is an ubiquitin-binding protein that binds to ubiquitinated protein aggregates and carries them to the autophagosomal membrane by directly binding to LC3. LC3 is detected as two bands: cytosolic LC3-I (18 kDa) and LC3-II (16 kDa), which is present in isolated membranes and autophagosomes and much less in autophagolysosomes. The amount of LC3-II is closely correlated with the amount of autophagosomes, however, since LC3-II is itself degraded by autophagy (in autophagolysosomes), cells were beforehand incubated with bafilomycin that prevents autophagosome-lysosome fusion and LC3-II turnover. Thicker LC3-II band can be then interpreted as corresponding to an increased autophagic flux.

**Figure 4:**
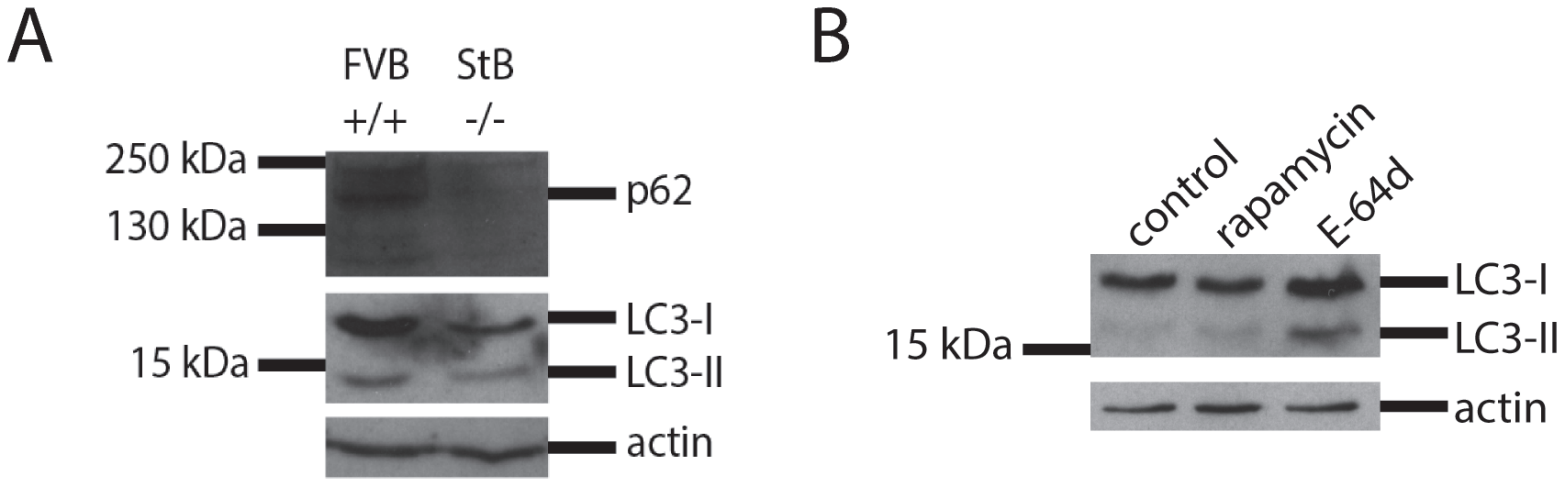
Western blot analysis (A) of the protein expression of p62 and LC3-I and LC3-II in primary FVB +/+ (wild type) and StB −/− (knock out) astrocytes. (B) Western blot analysis of the protein expression of LC3-I and LC3-II in primary stefin B KO astrocytes after incubation with cathepsin inhibitor. 20 nM rapamycin and 5 µM E-64d were added to the medium for 24 hours. Cells were incubated with 80 nM bafilomycin for 30 minutes.

**Figure 5:**
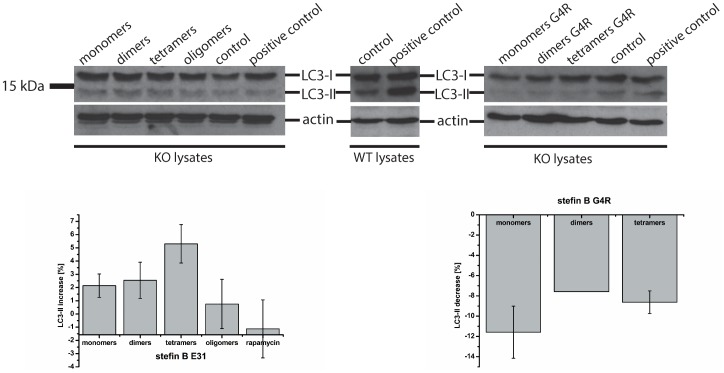
Western blot analysis of the protein expression of LC3-I and LC3-II in primary astrocytes after the addition of stefin B to the medium. Stefin B deficient (KO) astrocytes were incubated for 24 hours with different forms of the wild type (wt) stefin B (A) and G4R mutant (C) protein as stated on the image. Below each immunoblot a graphic representation of densitometric analysis of three (wt stefin B) (B) or two (G4R mutant) (D) independent experiments is shown. For positive control, 20 nM rapamycin was added for 24 hours. Cells were incubated with 80 nM bafilomycin for 30 minutes.

We measured autophagy levels of p62 and LC3 in wt and KO astrocytes. Western blot analysis showed that KO astrocytes had reduced p62 and LC3 levels compared with the wt cells which would indicate induced autophagy and aggregate clearance (Figure 4A). However, in the next experiments, bafilomycin was added to cells before preparing cell lysates. Bafilomycin is an inhibitor of the vacuolar H^+^-ATPase, thus preventing the fusion of autophagosomes with lysosomes and the degradation of the LC3-II protein. In this case, the immublotting showed that the KO cells had much decreased basal level of autophagy which could not be induced by rapamycin (Figure 5A, B).

In order to test how stefin B affects autophagy, different oligomers of wt stefin B were added to the medium of KO cell cultures. With the addition of stefin B monomers, dimers and tetramers, a slight increase in LC3-II band could be observed (Figure 5A, B) which was confirmed by densitometry. In addition, the effect of E-64d, an irreversible inhibitor of cathepsins, on LC3 processing was tested. E-64d increased the LC3-II band (Figure 4B) in agreement with a possible involvement of cathepsins in autophagy completion.

### Detection of stefin B dimers in cells and importance of their residual activity

**Figure 6:**
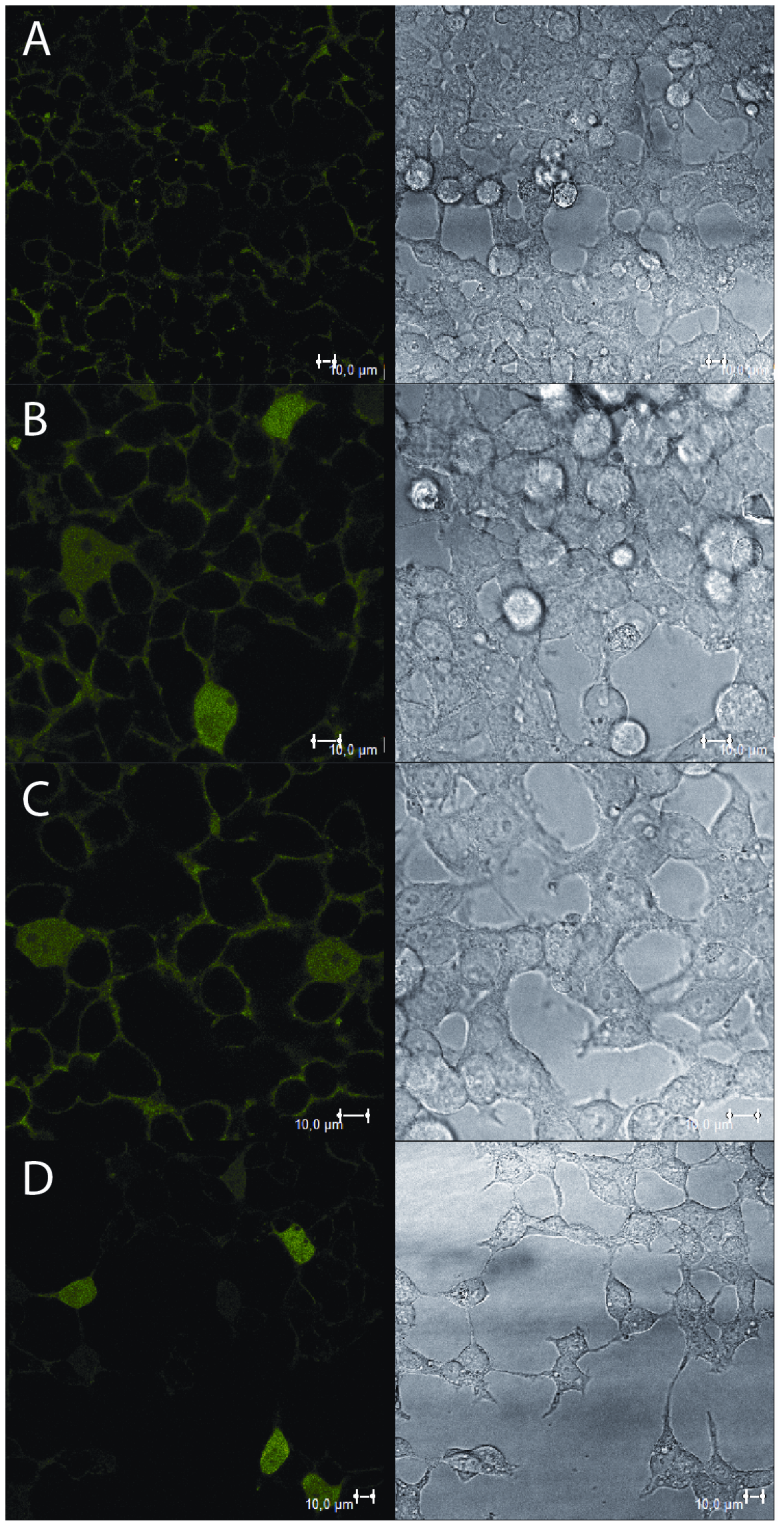
Bimolecular fluorescence microscopy - dimerization of stefin B in HEK293 cells. Cells were transfected with different combinations of plasmids to follow dimerization of over expressed proteins. For each protein a yellow fluorescent N-terminal (NYFP) or C-terminal half (CYFP), respectively, was attached to the C-terminus of the desired protein. A: control: wild type (WT)-NYFP + SUMO-CYFP; B: WT-NYFP + WT-CYFP; C: G4R-NYFP + G4R-CYFP; D: G4R-NYFP + WT-CYFP. SUMO protein was selected as the control protein due to its high solubility and abundance. Scale bar: 10 µm.

Stefin B readily forms dimers, tetramers and even higher oligomers *in vitro* [10], [13], [49] and formation of oligomers has been proven also in cells [15], [16]. Monomers of stefin B were already shown to be present in the nucleus [16], [17]. Here we confirm the existence of the dimeric form of the protein in both the cytoplasm and nucleus of HEK293 cells using bimolecular fluorescence (BiFC) (Figure 6). The wt protein interacted and formed mixed dimers with the G4R mutant, stefin B variant devoid of protease inhibitory activity but preserving a wt-like fold (Figure 6D). This mutant alone also formed intracellular dimers, however, the monomer/monomer interaction was weaker (Figure 6C). As a negative control, interaction of wt stefin B with SUMO protein was tested due to its high abundance and solubility. No intracellular fluorescence was detected, reporting thus on the specificity of the detected stefin B dimers (Figure 6A).

Due to its ability to oligomerize, the G4R mutant was additionally tested whether it can affect autophagy as the wt. On the contrary, the inhibitory inactive mutant caused a reduction in both LC3-I and LC3-II, which was confirmed by densitometry (Figure 5C, D). The underlying cause of this effect could arise from both the loss of inhibitory activity and the reported toxicity of the mutant [8].

### Searching for stefin B interactors in yeast model

In order to identify potential stefin B interactors, the wt protein (*CSTB* gene) was expressed in all the non-essential gene deletion yeast *Saccharomyces cerevisiae* strains and SDL analysis was performed. The growth rate of double mutants expressing human stefin B in the background of yeast gene deletions was measured. Results of the SDL experiment are shown in Tables 2 and 3.

**Table 2:** Yeast *S. cerevisiae* genes in positive genetic interaction with stefin B gene (cystatin B gene - *CSTB*).

Systematic (standard) name of the gene	Function of the corresponding protein	R
*YDR127W (ARO1)*	Penta-functional aromatic protein, catalysing 5 steps in synthesis of aromatic amino acids	13.893
***YLR324W (PEX30)***	Peroxisomal integral protein, involved in regulation of peroxisome number	12.558
***YML048W (GSF2)***	Integral membrane ER protein, binding misfolded proteins	10.597
*YKL132C (RMA1*	Putative dihydrofolate synthetase, contributing in mitochondrial synthesis binding nucleotides and ATP	7.416
***YGR122W***	A protein which likely regulates pH and binds to complex ESCRT-III, which sorts and transports ubiquinated proteins	6.754
*YOL086C (ADH1)*	Alcohol dehydrogenase, biosynthesis of alcohol via fermentation of glucose	3.736
*YAR031W (PRM9)*	Membrane protein regulated by pheromones, involved in vesicular transport	2.979
*YBR085W (AAC3)*	Mitochondrial transporter of ADP/ATP	2.836
*YPR054W (SMK1)*	MAP kinase, required for building the walls of spores	2.774
*YCR107W (AAD3)*	Putative aryl-alcohol dehydrogenase, involved in metabolism of aldehydes	2.645
*YPR040W (TIP41)*	Protein regulating phosholipase A2, involved in the signaling pathway of rapamycin	2.581
*YPR179C (HDA3)*	Subunit of a histone deacetylase complex	2.530
***YPR079W(MRL1)***	Membrane binding protein, potential receptor, involved in transport to vacuoles	2.327
*YPL262W (FUM1)*	Fumarate hydratase involved in the Krebs cycle	2.321
*YPR007C (REC8)*	Meiosis-specific component of sister chromatid cohesion complex	2.305
*YPR122W (AXL1)*	a-factor specific protease, involved in maturation of pheromones	2.352
***YOL147C (PEX11)***	Peroxisomal membrane protein required for proliferation and oxidation of fatty acids	2.201
*YFL013C (IES1)*	Subunit INO80, contributing to shift of chromatin within nucleosome	2.185

Functions of the encoded proteins according to SGD (http://www.yeastgenome.org/) are shown. R denotes relative growth rate compared to the reference strain (average of two biological replicates). Genes that have a function in vesicular transport are stressed in **bold**. Two proteins whose function is related to autophagy are also given in **bold**.

**Table 3:** Yeast *S. cerevisiae* genes in negative genetic interaction with stefin B gene (cystatin B gene - *CSTB*).

Systematic (standard) name of the gene	Function of the corresponding protein	R
*YBR267W (REI1)*	Cytosolic protein, involved in the large ribosome subunit biogenesis	0.381
*YCR086W (CSM1)*	Nucleolar protein mediating homolog segregation in meiosis	0.294
*YDR174W (HMO1)*	Protein involved in transcription and bending of dsDNA	0.406
*YDR378C (LSM6)*	Protein involved in mRNA processing and its cytosolic degradation	0.395
*YGR270W (YTA7)*	Chromatin protein regulating histones expression	0.314
*YHR081W (LRP1)*	Nuclear protein involved in DNA maintenance and RNA processing, related to exosomes	0.331
*YIL087C*	Mitochondrial protein with unknown function	0.429
*YIL128W (MET18)*	Component of cytosolic iron-sulfur protein assembly machinery	0.424
*YJR099W (YUH1)*	Ubiquitin C-terminal hydrolase	0.261
***YLL028W (TPO1)***	Polyamine transporter, recognizes spermine, spermidine and putrescine	0.414
*YLR410W (VIP1)*	Cytosolic kinase, important in biosynthesis of inositol phosphates	0.499
*YML032C (RAD52*	Nuclear protein involved in the repair of double-strand breaks in DNA	0.429
*YNL070W (TOM7)*	Component of the translocase of outer membrane complex	0.462

Functions of the encoded proteins according to SGD (http://www.yeastgenome.org/) are shown. R denotes relative growth rate compared to the reference strain (average of two biological replicates). A gene that has a function in polyamine transport is written in **bold** as well as a gene involved in inositol phosphates pathways, up-stream of autophagy.

Table 2 lists genes in positive genetic interaction with stefin B whose mutation resulted in a relatively faster growth rate. Apart from proteins involved in basic cellular metabolism of sugars, aromatic amino acids, aldehydes, Krebs cycle, oxidation of fatty acids and mitochondrial biosynthesis, a protein regulated by pheromones involved in vesicular transport, a histone deacetylase (related to autophagy [50]), a protein involved in the rapamycin signaling pathway and two peroxisomal proteins (autophagic receptors may also target peroxisomes [50]) were detected. Two genes involved in cellular response to misfolded proteins were also identified: i) an integral endoplasmatic reticulum (ER) membrane protein that binds misfolded proteins and ii) a protein involved in pH regulation and in sorting/transport of ubiquinated proteins.

Negative interactors, causing a decrease in the rate of yeast growth, are presented in Table 3. Apart from many nuclear proteins important for transcription, DNA repair and mRNA, a kinase involved in the inositol phosphates biosynthesis, an ubiquitin hydrolase and two additional membrane transport proteins were found: one regulates transport through mitochondrial membrane and another is a transport protein for polyamines (spermine).

## Discussion

### Higher amount of protein aggregates and reduced autophagy in KO cells

Primary stefin B KO astrocytes cultures prepared from stefin B KO mice were compared to primary wt astrocytes. Increased protein aggregation was observed in KO stefin B astrocytes (Figure 1A-G), suggesting a role of the protein in the prevention of protein aggregates accumulation. [16], [51], [52] [13], [18]It has been shown that the extracellular cystatin, cystatin C, induces autophagy [25]. We proposed previously that autophagy impairment could be related to PMEs and EPM1 [26]. To date, protein inclusions containing the lysosomal protein cathepsin B and CD68 or RNA-binding protein FUS were found only in one EPM1 patient with a dodecamer repeat expansion mutation [38], likely because other clinical studies did not address the presence of protein aggregates. No further changes were observed in the levels of protein aggregates in KO astrocytes upon addition of rapamycin (Figure 1G), which already pointed to a dysfunctional autophagy in these cells. In contrast, the number of aggregates decreased with the addition of stefin B monomers, dimers and tetramers to the culture medium (Figure 1B-E). The existence of oligomers of stefin B in cells has already been proven [15] and here we additionally show by BiFC that the wt and G4R indeed form dimers in the cytoplasm and in the nucleus (Figure 6B-D). Of note, all the oligomers and, of course, the monomers showed inhibitory activity towards cathepsins (Figure S1). Therefore, the mechanism of interference with autophagy flux could well be cathepsin dependent as the G4R inactive stefin B mutant did not increase the autophagic activity (Figure 5C, D).

The autophagic flux in primary astrocytes was monitored using p62 and LC3 as autophagy markers. Reduced levels of p62 and both LC3 bands could be observed in KO cells compared to wt cells (Figure 4A). Initially, these results suggested an increased autophagic flux in cells lacking stefin B. However, when adding bafilomycin, an inhibitor of H^+^-ATPase into the cell medium, autophagy levels of KO cells were clearly reduced compared to the wt and even the addition of rapamycin was not able to rescue them (Figure 5A, B). In our model, thus, autophagy may be initially induced, as indicated by the low p62 levels, however, a blockage must occur on the way to complete autophagic degradation. As suggested by Nixon [50], the initial induction of autophagy may act as a protective mechanism to fight the accumulation of protein aggregates. However, if the degradation process is not fully functional, it may become counterproductive and even contribute to the etiology of the disease.

The addition of different stefin B forms (monomers, dimers and tetramers) to the medium slightly increased the amount of LC3-II band (Figure 5A, B), meaning that stefin B could have a role in inducing autophagy. This effect seems to depend on its inhibitory activity since the addition of the inactive G4R mutant did not affect LC3 levels or even reduced them (Figure 5C, D). This view is in accordance with the fact that addition of E-64d, a generic irreversible inhibitor of cathepsins, increased the LC3-II band, apparently increasing autophagy (Figure 4B). However, this increase in LC3-II could also be a consequence of decreased lysosomal degradative potency due to massive cathepsin inhibition and not due to actual induction of autophagy.

Stefin B may also help to improve autophagic clearance in an indirect way: via its chaperone action, as proposed before [13], [18], by lowering burden of small aggregates from other proteins, or by mediating cytoskeleton building [53] and helping in vesicular transport.

Our results that stefin B positively influences autophagy are in contrast with the results published recently on an AD mouse model crossed with stefin B KO mice [39]. Yang and co-authors claimed that the stefin B deletion rescued autophagic-lysosomal pathology through increased cathepsin (protease) activity, whereas we show that its presence is connected to functional autophagy. They reported the presence of giant cathepsin D-positive compartments in the brain of transgenic mouse AD model. Cathepsin D was also detected in our MS analysis of the aggregates isolated from astrocytes lacking stefin B. Cathepsin D was found to be increased in other two mouse models of AD [54] and it was shown *in vitro* that it can cleave both tau, APP [55] and the Swedish mutant of APP [56], thus possibly generating the pathogenic Aβ42 fragment [57], [58]. However, the role of cathepsin D in AD is still controversial [59], [60]. Additionally, cathepsin D can cleave and inactivate cystatins (i.e. stefin A and B, cystatin C) [61], [62] *in vitro* and cystatin C in breast cancer microenvironment [63]. In this manner, cathepsin D can itself enhance the proteolytic activity of cysteine proteases. Interestingly, the same authors published a year before that cystatin C over expression rescued the stefin B KO phenotype by preventing the increase in cathepsin activation [64].

### Increased oxidative stress in KO cells

Stefin B KO cells displayed increased lipid peroxidation as compared to the wt and were more sensitive to peroxide treatment (Figure 2) which is in agreement with experiments done on neuronal cells [20], cancer cells [21] and activated macrophages (Maher et al., submitted).

A role of stefin B in protection against oxidative stress has been described [20] so that our results confirm a putative chaperone role of stefin B in preventing both protein aggregation and ROS production. What comes first - aggregates or ROS generation - remains unclear. It is possible that protein aggregates form first and cause increased ROS production due to membrane damage, or that increased ROS in absence of stefin B lead to increased protein aggregation. As the mechanism of stefin B activity on aggregates clearance is not resolved yet, we can only suspect the order of events. We suggest that stefin B is implied in autophagy itself and that toxic protein aggregates cannot be sufficiently cleared in stefin B KO astrocytes, leading to increased oxidative stress.

### TEM reveals several different proteinaceous and lipidous structures

TEM images of wt astrocytes (Figure 3A, B, Figure S2A) showed larger perinuclear structures of aggregated proteins, surrounded by a membrane that could be in some cases also observed by fluorescent microscopy (Figure 1F). These structures average 2.5 µm in diameter and resemble aggresomes that are intended to sequester protein aggregates for complete degradation by autophagy and are claimed to be neuroprotective [51]. Ultrastructure analysis of KO astrocytes revealed morphologically similar structures (Figure 3C, D) that were however smaller and had less complex composition compared to the wt cells. This could mean that the process of protein turnover would be more rapid and complete in KO cells. However, due to much increased oxidative stress in KO astrocytes compared to the wt cells we suggest that small protein aggregates diffusely accumulate in the cytoplasm of the KO cells and cannot be transported to microtubule-organizing center (MTOC).

In wt and KO astrocytes, many smaller vesicles of around 500 nm with internal membranes and relatively high protein content were distributed evenly through the cytoplasm (Figure S2E, F). These vesicles could be multivesicular organelles with acidic pH dedicated to normal proteolysis of cell proteins in their lumen [65]. In addition, in both cell types large organelle structures (size of around 3 µm), with exceedingly abundant multilamelar composition and limiting outer membrane could be observed (Figure S2B-D). Due to low protein content we cannot assure if protein aggregates are retained between the inner membranes of these large organelles. They could represent acidic vesicles in the terminal part of the endocytic pathway.

### Differences in the protein content of aggregates in wt and KO astrocytes

In order to compare the content of the protein aggregates of wt and KO cells LC-MS/MS analysis was performed (Table 1). In wt cells, tubulin and dynein were present, while they were absent or diminished in KO astrocytes. The absence of dyneins, motor proteins that move aggregates along microtubules, suggests that a step in aggregates' transport, accumulation into aggresomes and finally degradation could be impaired in KO cells. Interestingly, most chaperones and proteins of the ubiquitin proteasome system were not detected at all in the insoluble fraction of stefin B KO cells. A possible explanation would be that there is an insufficient protein misfolding response in the stefin B deficient cells, leading to protein aggregation or, that the aggregates would evade the protein quality machinery, including incomplete transportation and degradation pathways.

Furthermore, the MS analysis (Table 1) showed that the aggregates in KO cells structures had increased number of two lysosomal proteins, CD63 and cathepsin D. Cathepsin D was reported to be an important constituent of aggregates in a mouse AD model [39]. Also, cathepsin D polymorphisms are known to be associated with AD [66], however their effect was later on debated [67], [68], [69] or completely discarded [70], [71].

Fibronectin was increased in the insoluble fraction of KO cells and may be a result of glial activation as suggested by Lieuallen and co-workers [72], who found an increase in apoptosis and glial activation genes. Apolipoprotein D was suggested by the same authors to be involved in glial activation, however, in our experiments only apolipoprotein E was increased in stefin B KO astrocytes. Apoliprotein E defects and isoforms are associated with various neurodegenerative diseases, including AD. Similarly, the major prion protein was detected only in the aggregates of KO cells.

### Yeast studies agree with a possible role of stefin B in aggregate trafficking

In the yeast SDL study two genes involved in cellular response to misfolded proteins were identified. The first one corresponds to an integral ER membrane protein that binds misfolded proteins and the second one contributes in the sorting and transport of ubiquinated proteins. These two genes were in positive genetic interaction with stefin B, indicating that the proteins encoded are potential targets for the stefin B action in cells. Among the positive interactors (Table 2), there was also a gene involved in the rapamycin signaling pathway and among the negative interactors (Table 3) a gene encoding a transport protein for spermine and a kinase important in glycogen synthesis, which are all related to autophagy [50].

Results obtained in yeast support the possibility that stefin B is involved in protein misfolding response and contributes to the cellular transport of vesicles and cross-membrane transport of various proteins, including the autophagy inducers polyamines (spermine) [50].

### Conclusions

1. Increased protein aggregation, ROS generation and compromised autophagic flux were observed in stefin B KO astrocytes. The flux was improved by addition of stefin B monomers and other lower oligomers but not by rapamaycin. This suggests that the role of stefin B in removal of the aggregates and augmenting autophagy could be cathepsin dependent.

2. Mass spectrometry showed differences in composition in the insoluble fractions (aggregates) of wt and KO cells. KO samples lacked tubulins, dyneins, most chaperons and proteins of the ubiquitin proteasome system that indicated impairments in the aggregates transport mechanism and the quality control machinery, respectively. On the other hand, the insoluble fraction of KO cell showed increase in cathepsin D and CD63 (both of lysosomal origin), prion protein, serpins, fibronectin and apolipoprotein E.

3. TEM images show more abundant formation of aggresome-like structures in wt cells, while in KO cells small and scattered aggregates predominate.

4. The SDL screen in yeast agrees with the possibility that stefin B is involved in protein misfolding response, in cellular transport of vesicles and in membrane transport of various substances, including polyamines, which are autophagy inducers, and in glycogen synthesis, which comprises an important signaling pathway up-stream of autophagy.

5. Combined results thus confirm that stefin B could be involved in the cell's response to misfolded proteins: either together with other chaperones to reverse misfolding or, helping in lipid vesicles formation and transport needed towards the MTOC. By promoting aggregate clearance and autophagic flux, stefin B could indirectly reduce generation of ROS.

## Supporting Information

Figure S1 **BANA test - inhibitory activity of stefin B monomers, dimers, tetramers, and a mix of higher oligomers.** Average oligomeric species in the sample of higher oligomers were assumed as octamers. Inhibitory activity was measured colorimetrically at 520 nm after cleavage of the BANA substrate by papain. On the x axis, molar ratios [E]∶[I] are presented. Average values of two independent experiments are shown.
(TIFF)

Figure S2 **Transmission electron microscopy (TEM) of wild type (A, C, E) and KO (B, D, F) FVB mice astrocytes.** Cell proteins were contrasted with uranyl acetate, whereas membranes remained white (not contrasted) on ultrathin cell sections (70 nm). High content of aggregated proteins in large perinuclear structure is shown (A). *Open white arrow heads* point at three possible sites (invaginations) of the limiting outer membrane where new aggregated proteins may enter the structure (A). The positions of large organelles with exceedingly abundant membrane composition (w*hite arrows*, B, C, D), smaller 500-nm vesicles with high protein content and fewer membranes (*white arrow heads*, B, E, F), the nucleus (*, B), the mitochondria (*black arrow heads*, F) and the Golgi cisternae (*open black arrow heads*, B, E) are denoted. Estimated size of the large multilamelar vesicles (white arrows) is 3.7 µm in wt astrocytes (A) and 2.5 µm in KO astrocytes (B).
(TIF)

Table S1 **An extended and more detailed table of hits detected in wild type (wt) and stefin B knock out (KO) insoluble cell lysate fractions by LC-MS/MS.** Proteins that were found increased in KO or wt cells are denote in the sheets “upregulated” or “downregulated”, respectively (Supplied in Excel format).
(XLSX)
